# Supplementary material for: Blocking Dectin-1 prevents colorectal tumorigenesis by suppressing prostaglandin E2 production in myeloid-derived suppressor cells and enhancing IL-22 binding protein expression
Source: Nat Commun. 2023 Mar 17;14:1493. doi: 10.1038/s41467-023-37229-x (PMC10023663; doi:10.1038/s41467-023-37229-x)
Supplement: Supplementary file 1 — Supplementary Information [file 41467_2023_37229_MOESM1_ESM.pdf]

Supplementary Figures, Figure legends and Tables

Supplementary Figure 1

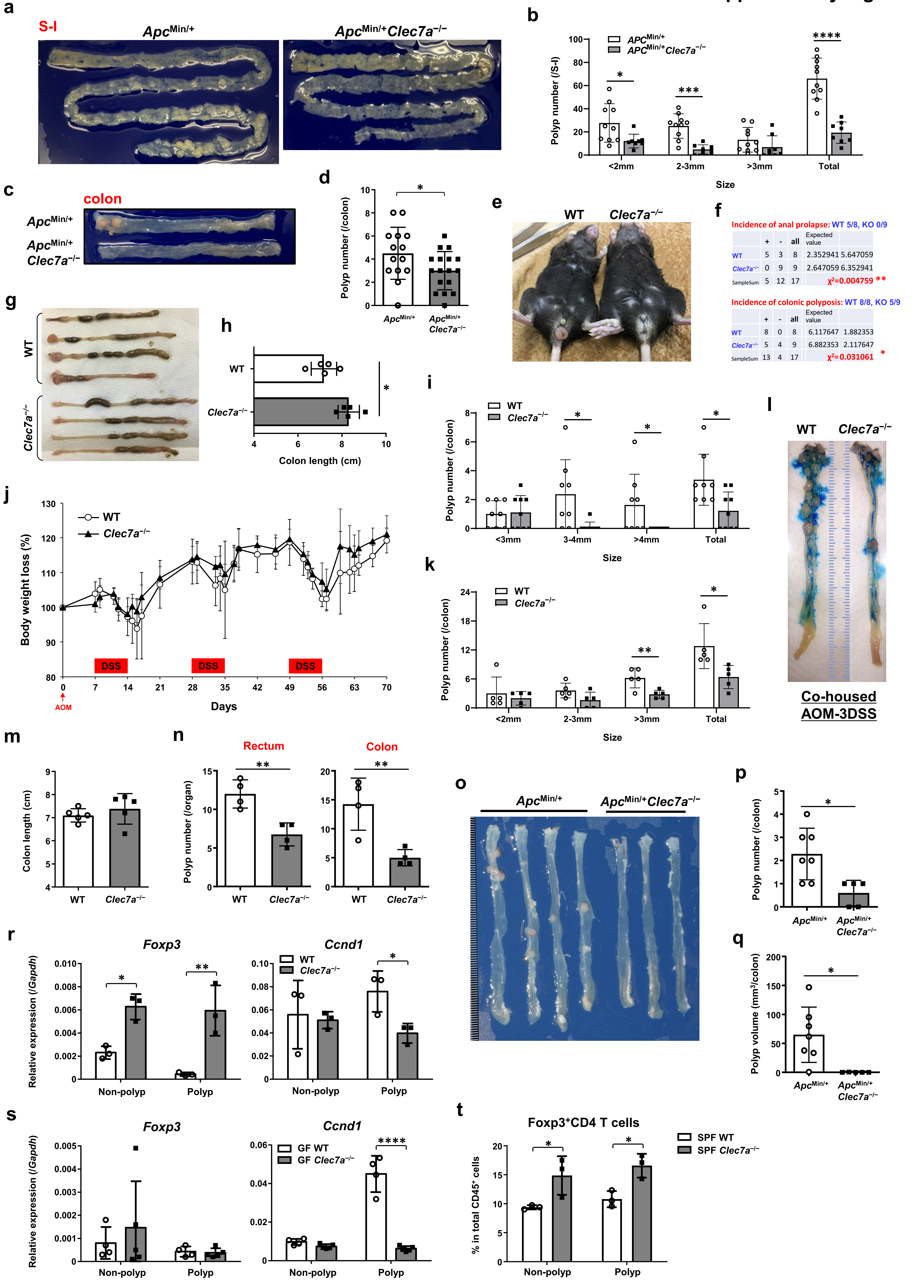

Supplementary Figure 1. Dectin-1-deficiency suppresses colorectal tumor, and this suppression is independent of commensal microbiota.

(a, b) *Apc*$^{Min/+}$ and *Apc*$^{Min/+}$*Clec7a*$^{-/-}$ mice were sacrificed at 20-23 weeks old. Gross observation of tumors in small intestine from indicated mice (a) and polyp numbers of indicated sizes (b; *P=0.023, ***P=0.0001, ****P<0.0001) are shown (n=10/*Apc*$^{Min/+}$ group, n=8/*Apc*$^{Min/+}$*Clec7a*$^{-/-}$ group).

(c, d) *Apc*$^{Min/+}$ and *Apc*$^{Min/+}$*Clec7a*$^{-/-}$ mice were sacrificed at 20-23 weeks old. Gross pathology of the colon (c) and number of colorectal polyps (d; *P=0.041) were examined (n=14/*Apc*$^{Min/+}$ group, n=17/*Apc*$^{Min/+}$*Clec7a*$^{-/-}$ group).

(e-i) WT and *Clec7a*$^{-/-}$ mice were administrated with AOM, and 7 days later, they were treated with 1% DSS for only 7 days, and normally housed for another 10 weeks before sacrifice (WT n=8, *Clec7a*$^{-/-}$ n=9). Anal prolapse are shown in WT mouse (e) and the incidence of prolapse and colorectal tumor were statistically evaluated (f). Colon length were measured (g, h; *P=0.0101), and the numbers of the rectal tumor of indicated sizes are shown (i; 3-4mm, *P=0.0128; >4mm, *P=0.0366; Total, *P=0.0114).

(j-n) WT and *Clec7a*$^{-/-}$ mice were co-housed after weaning at 4 weeks old, and 4 weeks later, they were treated with AOM-3DSS. Body weight loss of indicated mice was measured chronologically from the beginning of first DSS treatment as shown the x-axis (j). Mice were sacrificed at week 16 after AOM administration. Polyp numbers of indicated size (k; **P=0.0089, *P=0.0213) and in the colon and rectum (n; Rectum, **P=0.0044; Colon, **P=0.0078) were counted. Gross pathology of the colon was observed (l) and the colon length was also measured (m) after mice were sacrificed (j-m, n=5/group; n, n=4/group).

(o-q) *Apc*$^{Min/+}$ and *Apc*$^{Min/+}$*Clec7a*$^{-/-}$ mice were pre-treated with ABX cocktail VANM for 3 weeks. Then. they were administrated with 1.5% DSS + ABX for 1 week, followed by continuous treatment with ABX for another 4 weeks. Gross pathology of the colon (o), number of colorectal polyps (p, *Apc*$^{Min/+}$ n=7, *Apc*$^{Min/+}$*Clec7a*$^{-/-}$ n=5; *P=0.0113) and total polyp volume (q, *Apc*$^{Min/+}$ n=7, *Apc*$^{Min/+}$*Clec7a*$^{-/-}$ n=5; *P=0.0135) were examined.

(r) WT or *Clec7a*$^{-/-}$ mice were treated with AOM-3DSS and were sacrificed on week 16 after the first AOM-administration, and expression of indicated genes in colonic polyps and non-polyp tissues was determined by qPCR (n=3/group; *Foxp3*, *P=0.0199, **P=0.0030; *Ccnd1*, *P=0.0328).

(s) WT or *Clec7a*$^{-/-}$ GF mice were treated with AOM-3DSS and were sacrificed on week 36 after the first AOM-administration, and mRNA expression of indicated genes in colonic polyps and non-polyp tissues were determined by qPCR (GF WT n=4; GF *Clec7a*$^{-/-}$ n=5; ****P<0.0001).

(t) In the experiments described in (r), proportions of Foxp3^{+}CD4 T cells in total CD45^{+} cells from colorectal polyp or non-polyp tissues of SPF mice were examined by FACS analysis (n=3/group; Non-polyp, *P=0.0493; Polyp, *P=0.0386).

Data in (j-n) are representatives of three, and in (o-t) are representatives of two independent experiments. Data in (a-d) are pooled from three independent experiments. Data in (b, d, h-k, m, n, p-t) are expressed as means ± SD. Data in (b, d, h, i, k, n, p, q) are analyzed using unpaired two-tailed Student's *t*-test, in (f) using Chi-square test and in (r, s, t) using two-way ANOVA followed by Tukey's multiple-comparisons test. Source data are provided in the Source Data file.

Supplementary Figure 2

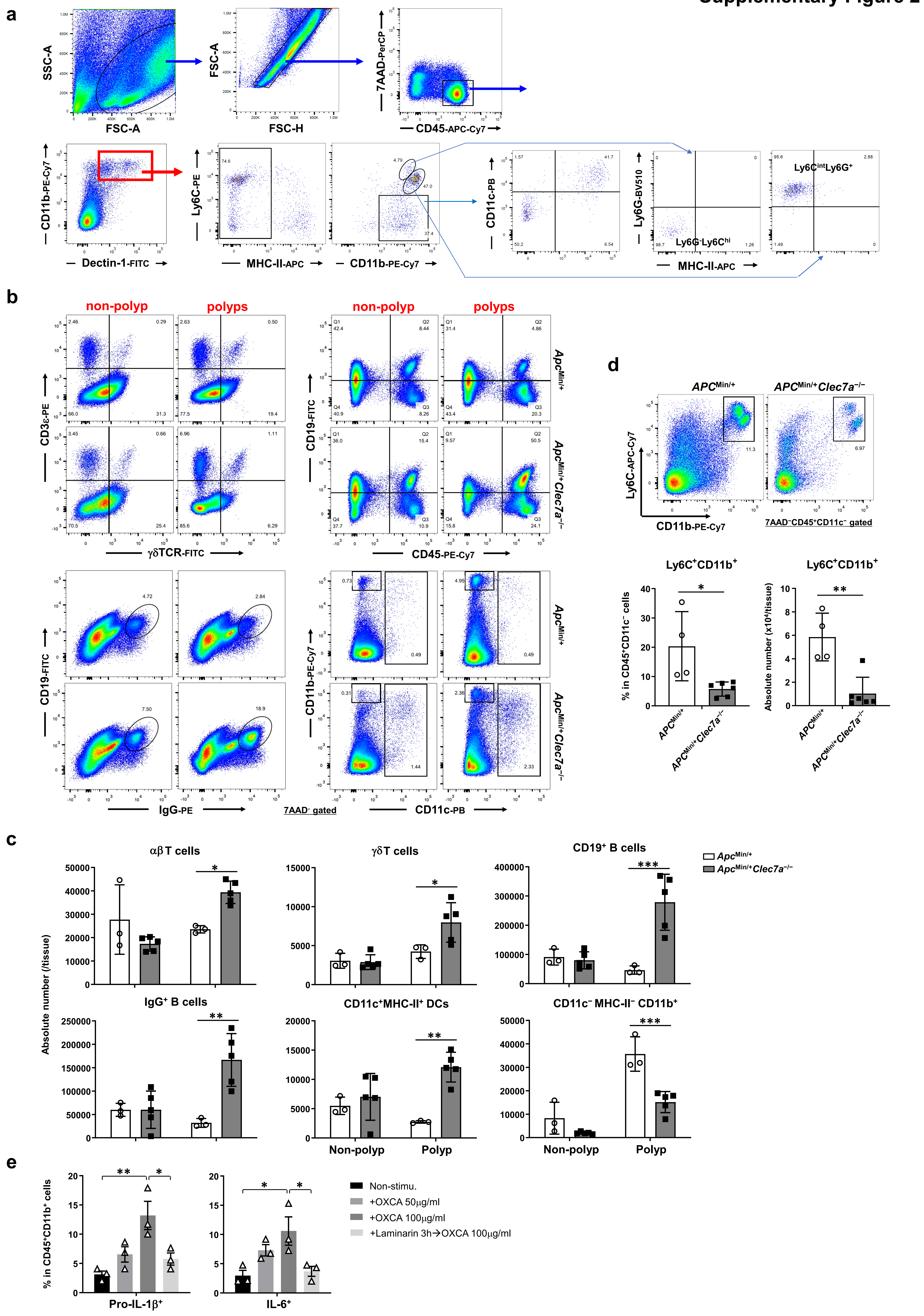

Supplementary Figure 2. The majority of Dectin-1-expressing cells in colorectal tumors are MHC-II$^-$ myeloid cells.

(a) In the experiment described in Fig. 2a, Dectin-1-expressing cell subsets infiltrating in colonic polyps were identified by flow cytometry (including gating strategy for usual analysis).

(b, c) In the experiment described in Fig. 2b, dot plot panels exhibiting the proportions (b) and numbers (c; αβT cells, *P=0.0374; γδT cells, *P=0.0393; CD19$^+$ B cells, ***P=0.0008; IgG$^+$ B cells, ***P=0.0032; MHC-II$^+$CD11c$^+$ DCs, **P=0.0031; CD11c$^-$MHC-II$^-$CD11b$^+$, ***P=0.0004) of T cells, B cells and DCs in tumor infiltrating cells were examined by flow cytometry.

(d) The same experiment described in Fig. 2b was carried out, and dot plot panels of the FACA analysis and histograms exhibiting the proportions and numbers of Ly6C$^+$CD11b$^+$ cells in tumors from indicated mice are shown (*Apc*$^{Min/+}$ n=4, *Apc*$^{Min/+}$*Clec7a*$^{-/-}$ n=6; *P=0.0163, **P=0.0020).

(e) In the experiment described in Fig. 2e, CD11b$^+$ and CD11c$^+$ cells were stimulated with OXCA or OXCA + laminarin for 6 h, then intracellular IL-6$^+$ and pro-IL-1β$^+$ cells were detected by flow cytometry (n=3 biologically independent samples/group; Pro-IL-1β$^+$ **P=0.0061, *P=0.0322; IL-6$^+$, 1st *P=0.0240, 2nd *P=0.0394).

Data in (a-e) are representatives of two independent experiments. Data in (c-e) are expressed as means ± SD. Data in (c) are analyzed using two-way ANOVA followed by Tukey's multiple-comparisons test, in (d) using unpaired two-tailed Student's *t*-test and in (e) using one-way ANOVA followed by Tukey's multiple-comparisons test. Source data are provided in the Source Data file.

Supplementary Figure 3

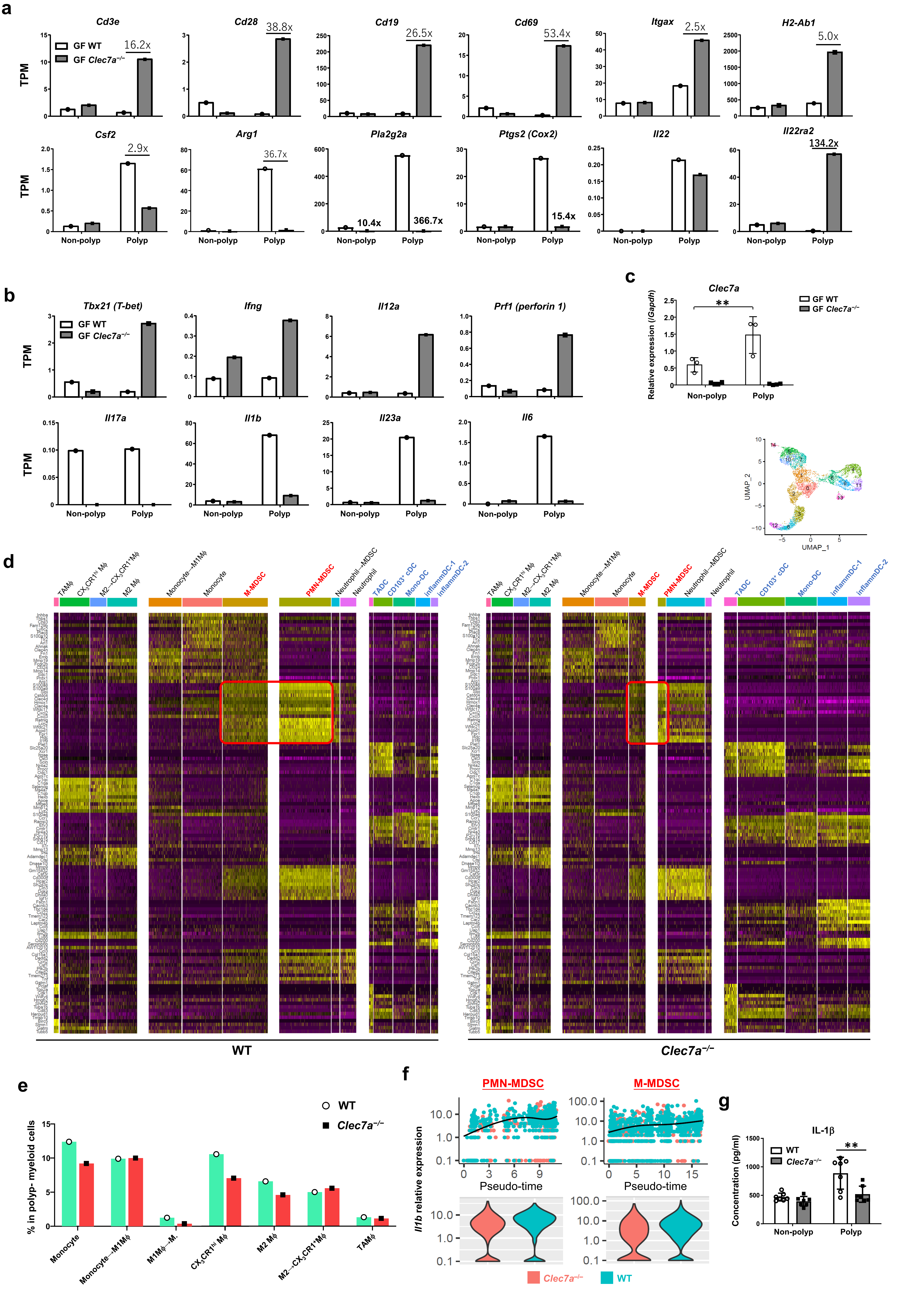

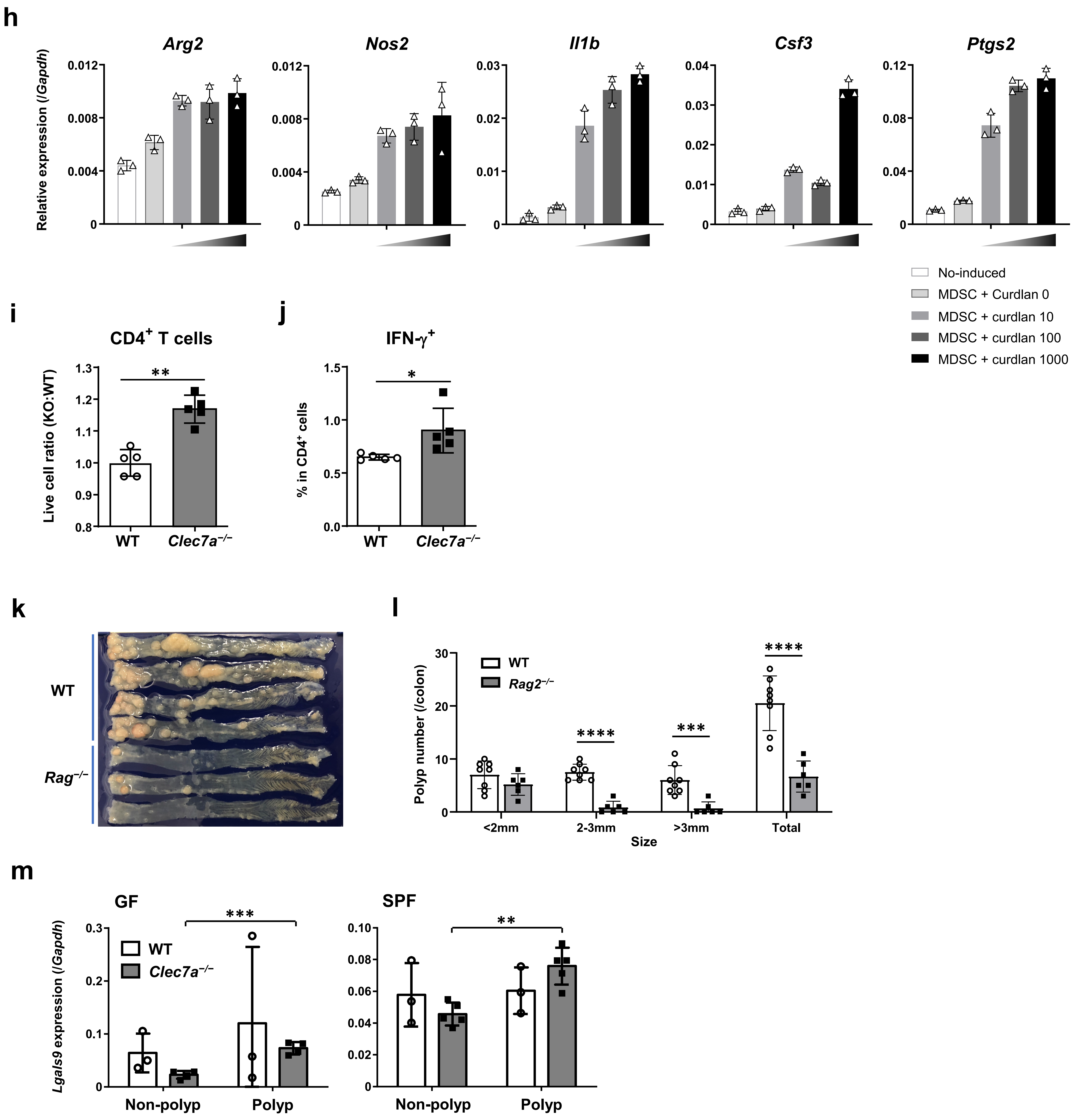
h
Arg2
Nos2
Il1b
Csf3
Ptgs2
Relative expression (/Gapdh)
No-induced
MDSC + Curdlan 0
MDSC + curdlan 10
MDSC + curdlan 100
MDSC + curdlan 1000
i
CD4+ T cells
Live cell ratio (KO:WT)
WT
Clec7a−/−
j
IFN-γ+
% in CD4+ cells
k
WT
Rag−/−
l
WT
Rag2−/−
Polyp number (/colon)
<2mm
2-3mm
>3mm
Total
Size
m
GF
SPF
WT
Clec7a−/−
Lgals9 expression (/Gapdh)
Non-polyp
Polyp

Supplementary Figure 3. Expression of most genes associated with anti-tumor immunity and MDSC population are changed in Dectin-1 deficient hosts.

(a, b) In the experiment described in Fig. 3a-d, the expression of selected genes encoding immune cell markers and tumor-associated factors (a) and of genes associated with Th1 or Th17 cells (b) in colonic polyps and non-polyp tissues was determined by RNA-seq analysis (4 WT and 5 *Clec7a*$^{-/-}$ GF mice pooled).

(c) The expression of *Clec7a* in colonic polyp and non-polyp tissues from GF WT and *Clec7a*$^{-/-}$ mice was examined by RT-qPCR (e, WT n=3, *Clec7a*$^{-/-}$ n=4; **P=0.0093).

(d-f) In the experiments described in Fig. 3e-h, scRNA-seq was carried out. The heatmap for top typical gene expression of each tumor-infiltrating myeloid cells and the UMAP (d), the proportions of monocyte and macrophage subsets in WT and *Clec7a*$^{-/-}$ mice (e) and *Il1b* expression changes during MDSC development (f) are shown (5 WT and 4 *Clec7a*$^{-/-}$ mice pooled).

(g) WT and *Clec7a*$^{-/-}$ mice were treated with AOM-3DSS for 16 weeks, and IL-1β concentration in colorectal polyps and non-polyp tissues was determined by ELISA (n=7/group, **P=0.0018).

(h) In the experiment described in Fig. 3i, after *in vitro* culture for 5 days, the expression of typical MDSC-specific and MDSC-promoting genes was examined by qPCR (n=3 biologically independent samples/group).

(i, j) In the experiment described in Fig. 3k, splenic Gr1^{+} cells from AOM-3DD-treated mice were isolated and were co-cultured with WT splenic T cells for 72 h. Numbers of live CD4^{+} T cell cultured with WT and *Clec7a*$^{-/-}$ Gr1^{+} cells was measured and the ratio of *Clec7a*$^{-/-}$ vs WT is shown (i; **P=0.0020). Cells were further restimulated with PMA + Ionomycin for 4 h, and the proportion of IFN-γ-producing cells in whole CD4^{+} T cells was determined by flow cytometry (j; *P=0.0289) (n=5 biologically independent samples/group).

(k, l) WT and *Rag2*$^{-/-}$ mice were i.p. administrated with AOM, and 7 days later, they were treated with 1% DSS for 3 cycles described in Methods. After 12 weeks of colonic tumor induction, these mice were sacrificed, and gross observation of colorectal tumors (k) and polyp number counting (l; ***P=0.0008, ****P<0.0001) were carried out (WT n=8, *Rag2*$^{-/-}$ n=6).

(m) In the experiment described in Fig. 1m-o, the expression of *Lgals9* encoding potential endogenous Dectin-1 ligands was determined by qPCR (GF WT n=3, *Clec7a*$^{-/-}$ n=4, left panel). After indicated SPF mice were administrated with AOM-3DSS for 16 weeks, the expression of *Lgals9* was examined by qPCR (WT n=3, *Clec7a*$^{-/-}$ n=5, right panel; ***P=0.0003, **P=0.0011).

Data in (c, g-m) are representatives of two independent experiments and are expressed as means ± SD. Data in (c, g) are analyzed using two-way ANOVA followed by Tukey's multiple-comparisons test and in (i, j, l, m) using unpaired two-tailed Student's *t*-test. Source data are provided in the Source Data file.

Supplementary Figure 4

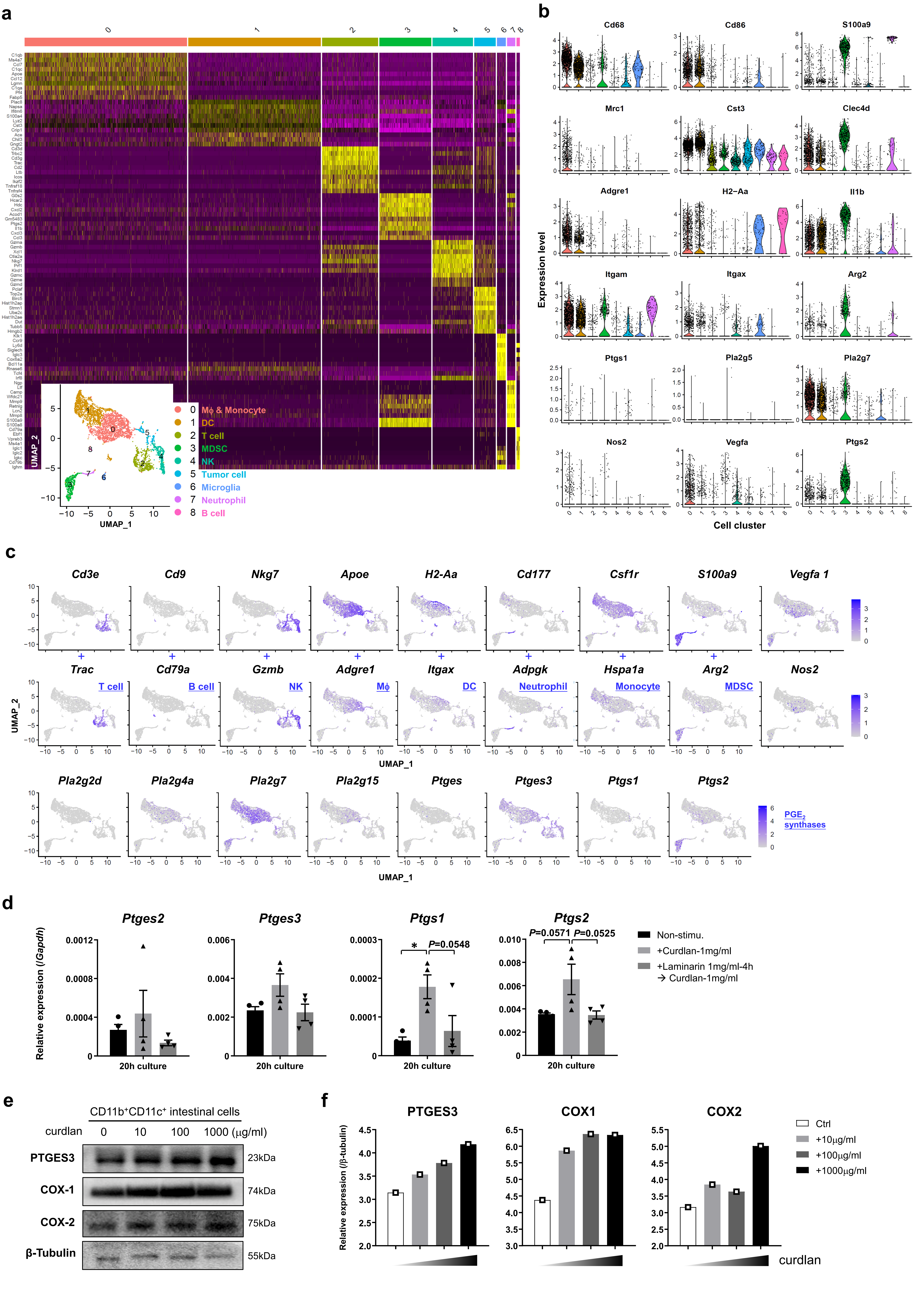

g

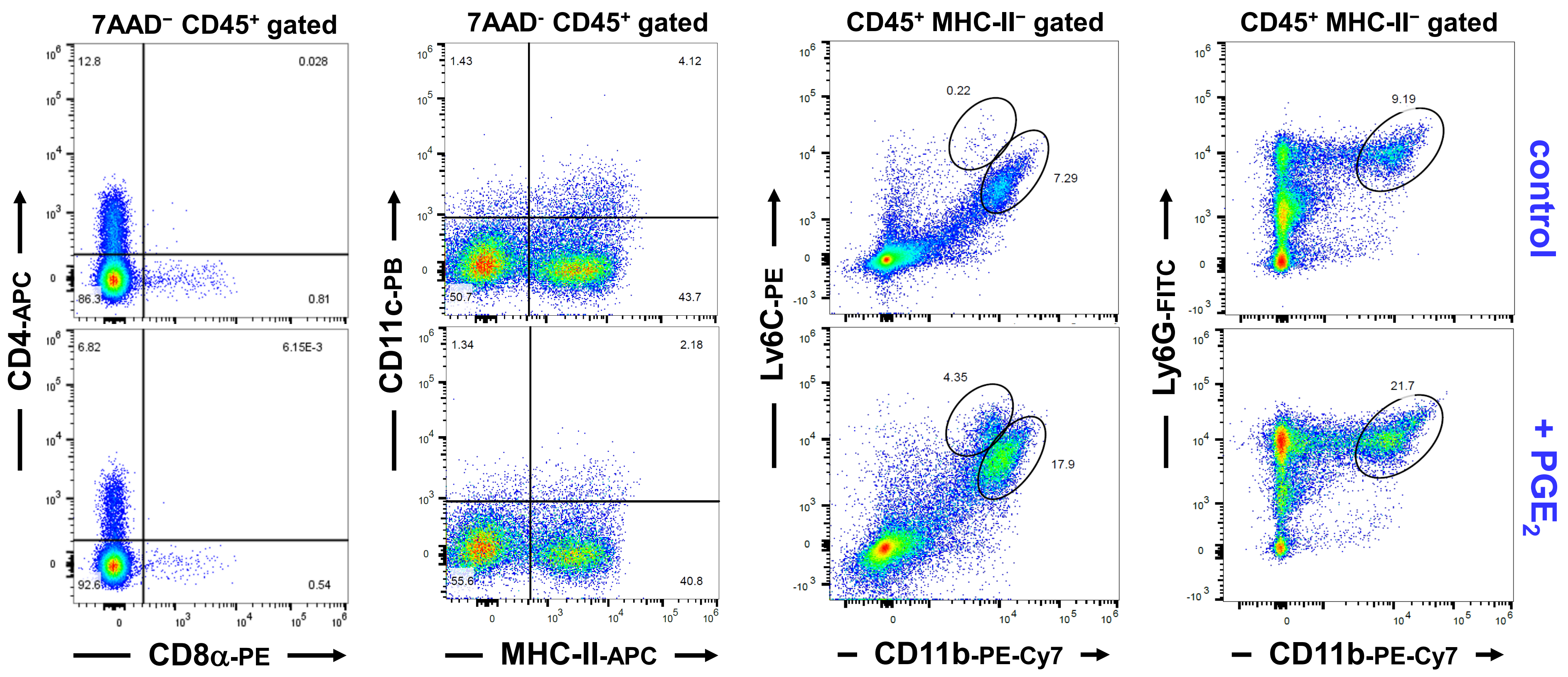
7AAD⁻ CD45⁺ gated
7AAD⁻ CD45⁺ gated
CD45⁺ MHC-II⁻ gated
CD45⁺ MHC-II⁻ gated
CD4-APC
CD11c-PB
Ly6C-PE
Ly6G-FITC
CD8α-PE
MHC-II-APC
CD11b-PE-Cy7
CD11b-PE-Cy7
control
+ PGE2
12.8
0.028
0.81
6.82
6.15E-3
0.54
1.43
4.12
43.7
1.34
2.18
40.8
0.22
7.29
4.35
17.9
9.19
21.7

h

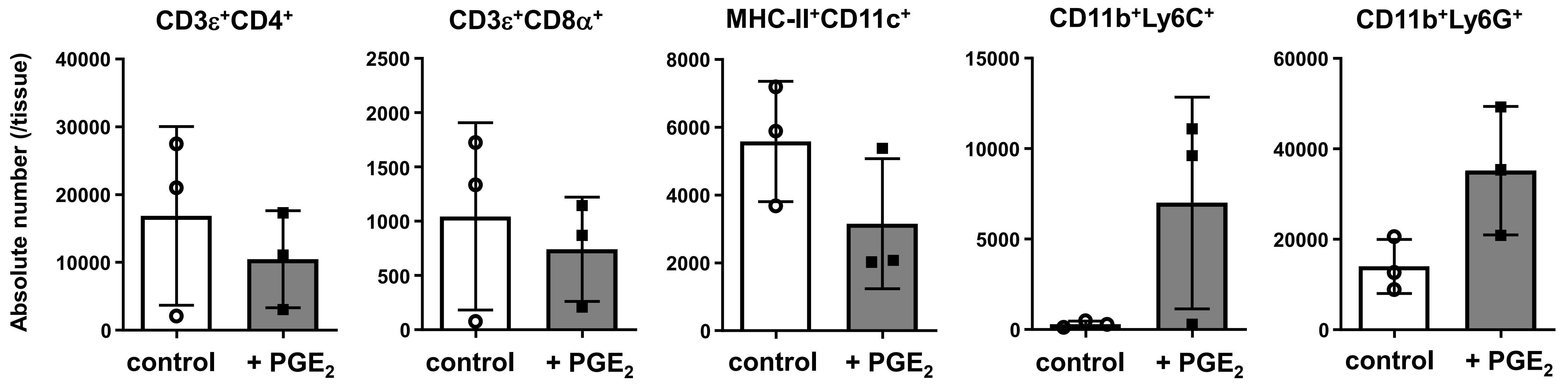
CD3ε⁺CD4⁺
CD3ε⁺CD8α⁺
MHC-II⁺CD11c⁺
CD11b⁺Ly6C⁺
CD11b⁺Ly6G⁺
Absolute number (/tissue)
40000
30000
20000
10000
0
2500
2000
1500
1000
500
0
8000
6000
4000
2000
0
15000
10000
5000
0
60000
40000
20000
0
control
+ PGE2

Supplementary Figure 4. PGE_2 synthases, especially COX1/2, are regulated by Dectin-1 signaling.

(a-c) To confirm the results obtained in Fig. 3e-h, another scRNA-seq analysis was performed by using a public data set (GSE196054), in which purified tumor-infiltrating $CD45^+$ cells from AOM-DSS treated C57BL/6 mice were analyzed. Heatmap (a), violin plots (b), and UMAPs (c) in which the top typical genes of each identified cell clusters, expression levels and distribution of cell-type-specific signature genes and of PGE_2 synthases in each cluster are exhibited respectively.

(d) Total polyp-infiltrating cells from $Apc^{Min/+}$ mice were harvested and were stimulated with curdlan for 20 h. Then, the expression of genes involved in PGE_2 synthesis was determined by RT-qPCR. Laminarin treatment was carried out for 4 h before treatment with curdlan (n=4 biologically independent samples/group; *Ptgs1*, *P=0.0218).

(e-f) $CD11b^+$ and $CD11c^+$ myeloid cells from colonic polyps of AOM-3DSS-treated WT mice were purified and were stimulated with curdlan. After 20h of culture, cells were harvested and PGE_2 synthase proteins were detected by Western-blot analysis.

(g) Dot plot panels of the flow cytometric analysis shown in Figure 5c.

(h) Absolute numbers of indicated cell subsets shown in Figure 5c (n=3 biologically independent samples/group).

Data in (d-h) are representatives of two independent experiments and in (d, h) are expressed as means ± SD. Data in (d) are analyzed using one-way ANOVA followed by Tukey's multiple-comparisons test. Source data are provided in the Source Data file.

Supplementary Figure 5

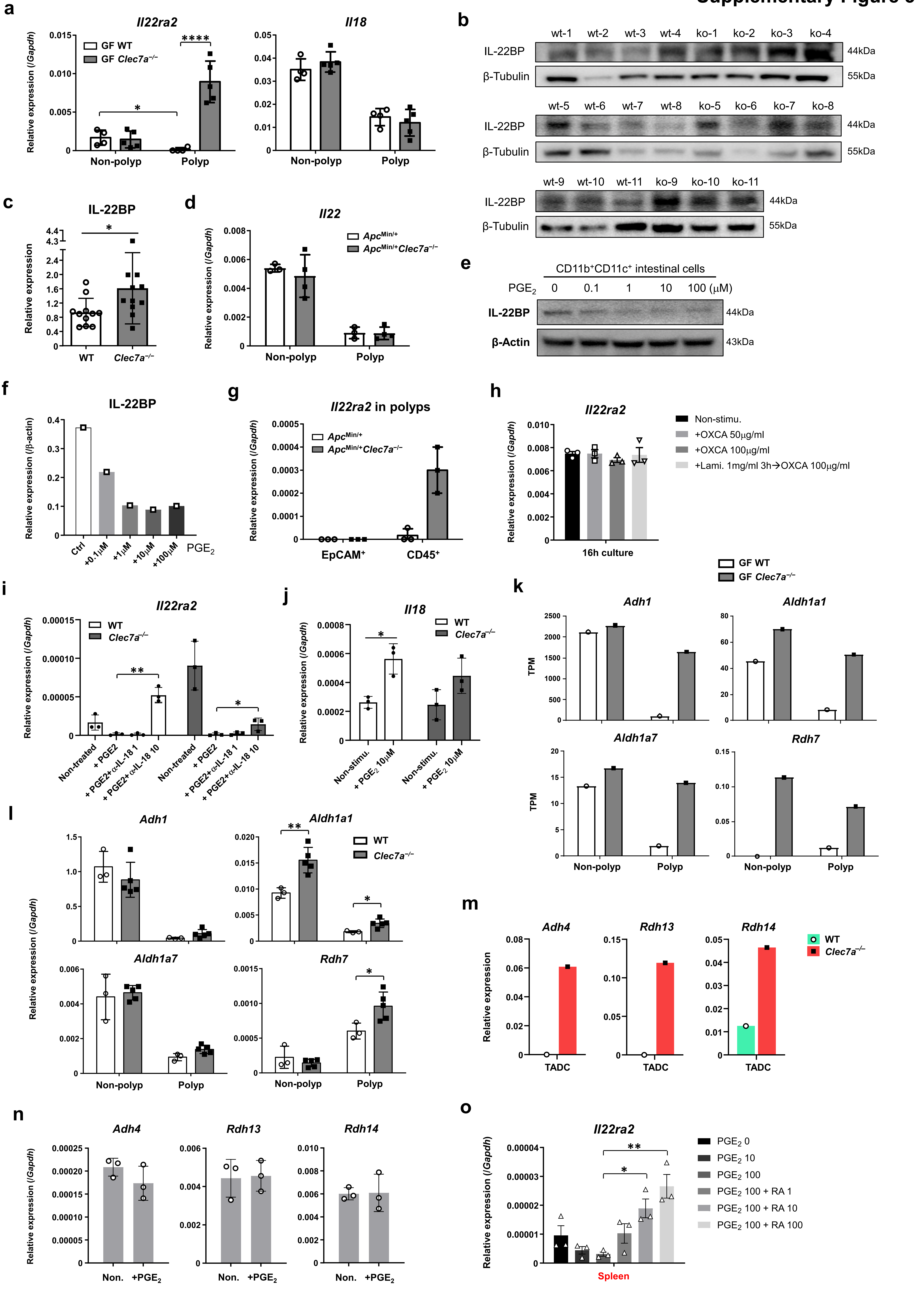

Supplementary Figure 5. Retinoic acid (RA) synthases are indirectly regulated by Dectin-1 signaling through PGE_2.

(a) WT or *Clec7a*$^{-/-}$ GF mice were treated with AOM-3DSS and were sacrificed on week 36 after the first AOM-administration. Messenger RNA expression of *Il22ra2* and *Il18* was determined by qPCR (a) (WT n=4, *Clec7a*$^{-/-}$ n=5; *P=0.022, ****P<0.0001).

(b, c) WT and *Clec7a*$^{-/-}$ mice were treated with AOM-3DSS, and 16 weeks later, colorectal polyps were harvested and IL-22BP was detected by Western blot analysis (n=11/group; *P=0.0497).

(d) In the experiment described in Fig. 6a, mRNA expression of *Il22* gene in colonic polyps and non-polyp tissues was determined by qPCR.

(e, f) $CD11b^+$ and $CD11c^+$ cells isolated from intestinal polyps of *Apc*$^{Min/+}$*Clec7a*$^{-/-}$ mice at 21 weeks old were cultured *in vitro* and were treated with indicated doses of PGE_2 for 20h, and IL-22BP expression was determined by Western blot analysis.

(g) Cells in intestinal polyps were harvested from *Apc*$^{Min/+}$ and *Apc*$^{Min/+}$*Clec7a*$^{-/-}$ mice at 23 weeks old, and *Il22ra2* expression was examined by RT-qPCR after separation of epithelial cells and leukocytes with autoMACS (n=3 biologically independent samples/group).

(h) C57BL/6J mice were treated with AOM-3DSS. After 11 weeks of AOM administration, these mice were sacrificed and $CD11b^+$ and $CD11c^+$ mixed cells were purified from colorectal polyps. Then, these cells were stimulated with OXCA, and *Il22ra2* expression was determined by RT-qPCR. In the last experimental group, laminarin treatment was carried out for 3 h before OXCA treatment (n=3 biologically independent samples/group).

(i) $CD11b^+$ and $CD11c^+$ cells isolated from the colon of *Apc*$^{Min/+}$ and *Apc*$^{Min/+}$*Clec7a*$^{-/-}$ mice were treated with 10μM PGE_2 for 20h in the present of anti-IL-18 antibody (1 or 10 μg/ml), and *Il22ra2* expression was determined by qPCR (n=3 biologically independent samples/group; **P=0.0031, *P=0.0264).

(j) $CD11b^+$ and $CD11c^+$ cells isolated from colon of *Apc*$^{Min/+}$ and *Apc*$^{Min/+}$*Clec7a*$^{-/-}$ mice were treated with 10μM PGE_2 for 20h, and *Il18* expression was determined by qPCR (n=3 biologically independent samples/group; *P=0.0232).

(k) WT or *Clec7a*$^{-/-}$ GF mice were treated with AOM-3DSS and were sacrificed on week 36 after the first AOM-administration. Messenger RNA expression of genes encoding RA-synthesizing enzymes was determined by RNA-seq.

(l) Total RNA of polyps or non-polyp intestinal tissues from SPF WT and *Clec7a*$^{-/-}$ mice administrated with AOM followed by 3 cycles of DSS for total 16 weeks were harvested and the expression of indicated genes was examined by qPCR (WT n=3, *Clec7a*$^{-/-}$ n=5; *Aldh1a1*, **P=0.0059, *P=0.0169; *Rdh7*, *P=0.0248).

(m) In the experiment described in Fig. 6m, mRNA expression of genes encoding other RA synthases was determined by scRNA-seq.

(n) In the experiment described in Fig. 6n, mRNA expression of genes encoding other RA synthases was determined by qPCR (n=3 biologically independent samples/group).

(o) $CD11b^+$ and $CD11c^+$ cells isolated from WT mouse spleen were co-treated with indicated doses of PGE_2 and RA for 20h, and expression level of *Il22ra2* was examined by qPCR (n=3 biologically independent samples/group; *P=0.0243, **P=0.0012).

Data in (a, d, e, g, h, i, j, l, n, o) are representatives of two independent experiments and in (a, c, d, g-j, l, n, o) are expressed as means ± SD. Data in (a, i, l) are analyzed using two-way ANOVA followed by Tukey's multiple-comparisons test, in (c) using unpaired two-tailed Student's *t*-test and in (o) using one-way ANOVA followed by Tukey's multiple-comparisons test. Source data are provided in the Source Data file.

Supplementary Figure 6

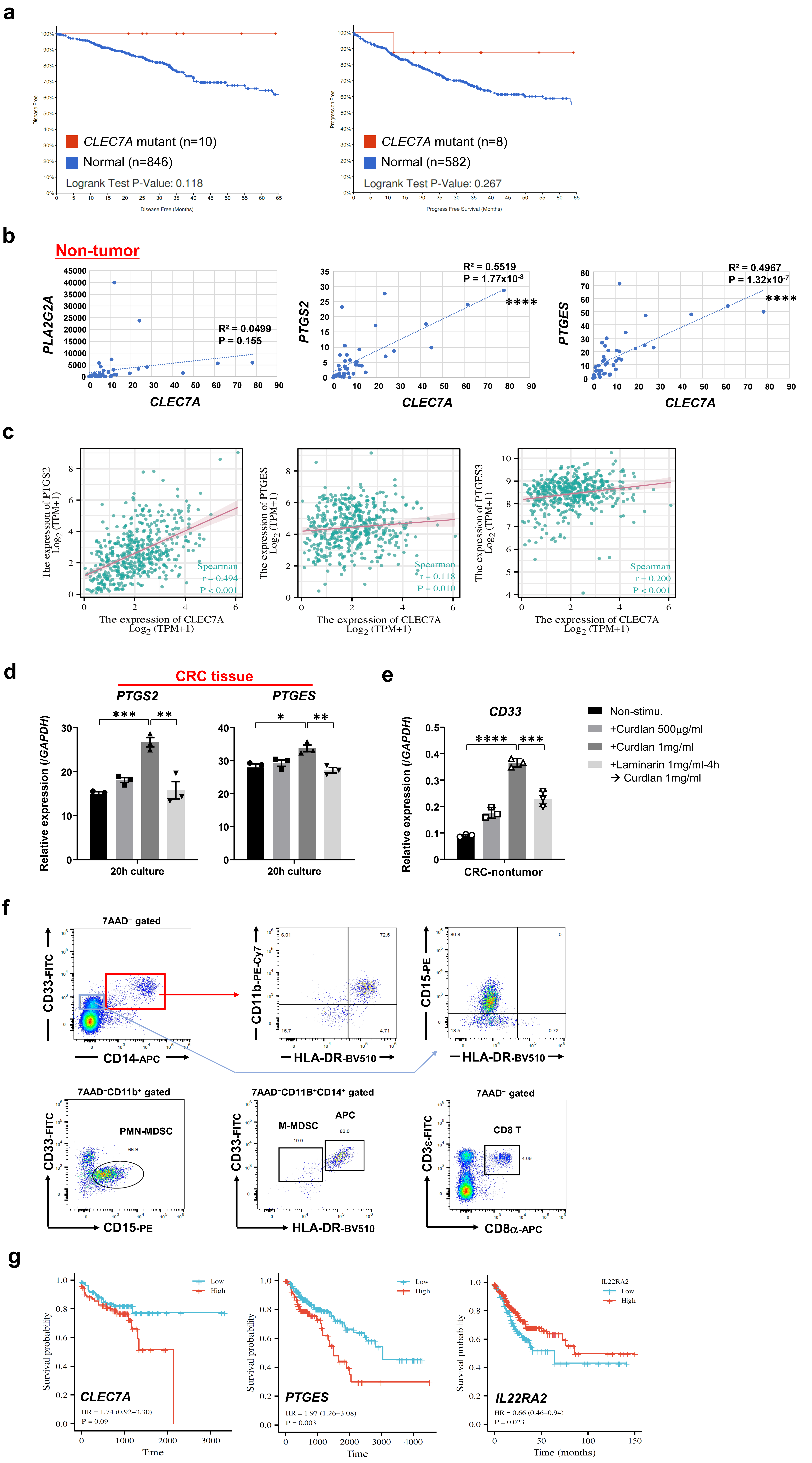

Supplementary Figure 6. The expression of PGE_2-synthesizing enzymes is induced by DECTIN-1 signaling in CRC patients.

(a) Survival rates of disease free (left panel) and progression free (right panel) in CRC patients with *CLEC7A* gene mutant or normal control were compared by analyzing public data in TCGA database.

(b) Non-tumor specimens of total 44 CRC patients were collected and correlations of the expressions between PGE_2-synthesizing enzyme and DECTIN-1 are shown.

(c) The correlations between expression levels of *CLEC7A* and indicated genes encoding PGE_2 synthases were examined by using the public data in TCGA database.

(d) Fresh CRC specimens were collected after surgery, and small pieces of the tumor tissue were cultured and stimulated with curdlan for 20 h. In the case of laminarin treatment, tissues were first treated with laminarin for 4 h, then curdlan was added to the culture. The expression of PGE_2-synthesizing enzymes was determined by qPCR. Data are the means ± SD of 3 wells (*PTGS2*, ***P=0.0005, **P=0.0037; *PTGES*, *P=0.0122, **P=0.0056).

(e) In the experiment described in Figure 7j, the expression of *CD33* in non-tumor tissue from CRC patients was examined by qPCR Data are the means ± SD of 3 wells (***P=0.0001, ****P<0.0001).

(f) Gating strategy of the flow cytometry analysis in the experiment described in Figure 7l and m.

(g) According to the expression levels of *CLEC7A*, *PTGES* and *IL22RA2* in tumors, CRC patients were classified into high and low groups using public database TCGA, and the survival rates of these CRC patients were compared (n=173 in *CLEC7A* panel, n=326 in *PTGES* panel, n=120 in *IL22RA2* panel).

Data in (d, e, f) are representatives of two independent experiments. Data in (a, g) are analyzed using Mantel-Cox log-rank test, in (b) using Pearson's correlation coefficient (r) test, in (c) using Spearman rank correlation test and in (d) using one-way ANOVA followed by Tukey's multiple-comparisons test. Source data are provided in the Source Data file.

Supplementary Figure 7

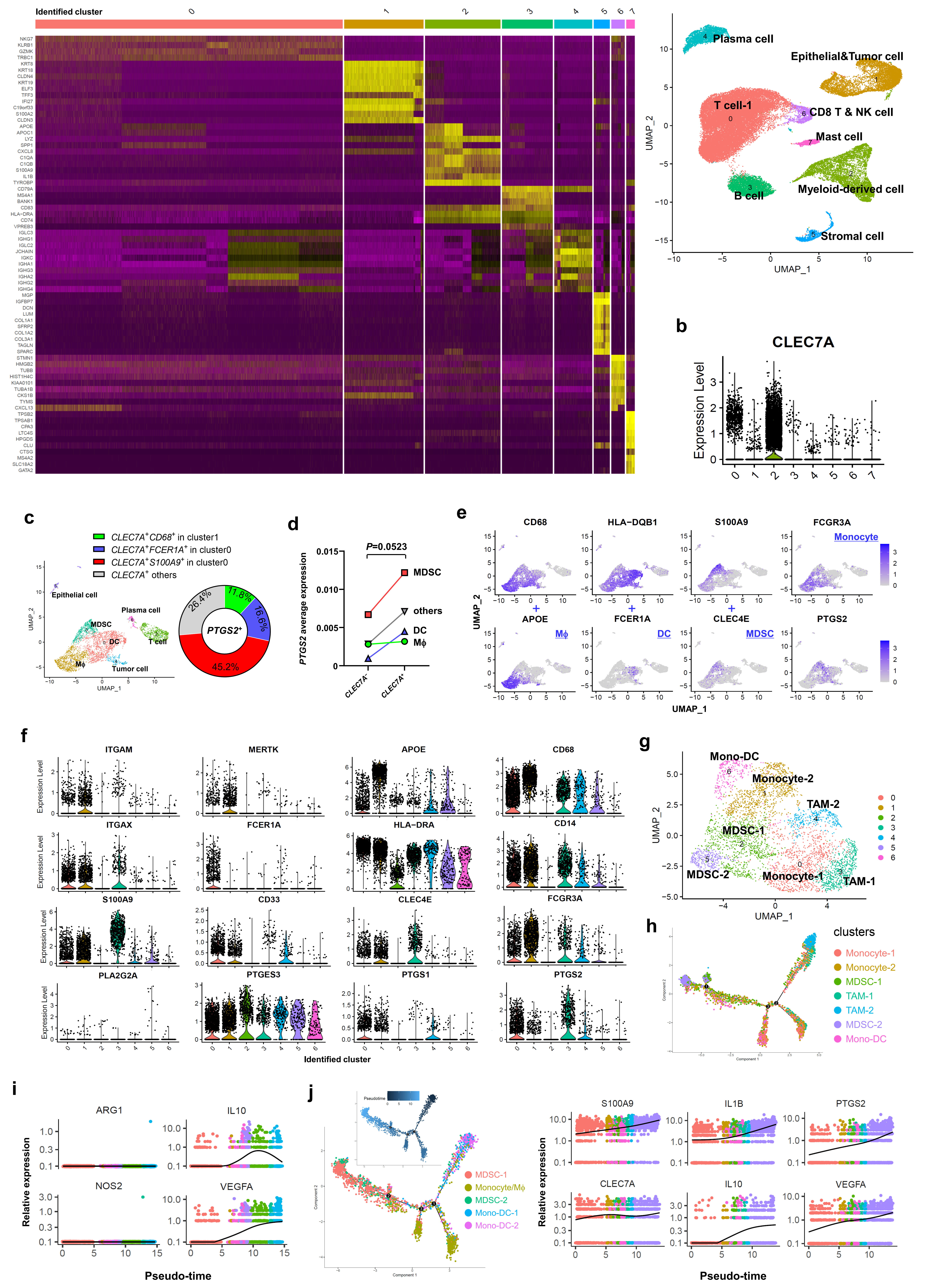

Supplementary Figure 7. ***CLEC7A*** **is highly expressed on MDSCs in human CRC.**

(a, b) Heatmap (a) and violin plot (b) obtained from single cell RNA-seq analysis by using pooled cell sequence datasets from six CRC patients (GSE178318) showing the expression level of top typical genes and *CLEC7A* in each identified cell cluster of CRC.

(c) The proportions of $CLEC7A^{+}PTGS2^{+}$ subpopulations in human CRC-associated cells obtained by scRNA-seq analysis are shown as a pie chart.

(d) Average expression levels of *PTGS2* in indicated cell types of $CLEC7A^{+}$ or $CLEC7A^{-}$ populations were examined by scRNA-seq analysis.

(e) UMAPs showing the distribution of indicated gene markers of MDSC (*CLEC4E*, *S100A9*), macrophage (*APOE*, *CD68*), DC (*FCER1A*, *HLA-DQB1*), monocyte (*FCGR3A*) and *PTGS2* in each $CLEC7A^{+}$ cell cluster in CRCs.

(f) Violin plots showing the expression levels of indicated genes representing macrophages (*ITGAM*, *MERTK*, *MRC1*, *CD68, APOE*), DCs (*ITGAX*, *FCER1A, HLA-DRA*), monocytes (*CD14*, *FCGR3A*), MDSCs (*S100A9*, *CD33*, *CLEC4E*) and PGE_2-synthases in each cluster from CRC-associated *CLEC7A*-expressing cells.

(g-i) After the extraction of myeloid cell cluster #2 from the whole population described in (a), $PTPRC^{lo}$ non-leukocytes, $CD3D^{+}TRAC^{+}$ T cells and $CST3^{hi}HLA\text{-}DRA^{hi}$ DCs were excluded from this cluster and rest of the cells were applied to pseudotime/ trajectory analysis. UMAP and violin plots show the annotation of each cell cluster applied for the analysis and the expression levels of myeloid cell-associated genes in each cluster, respectively (g). Pseudotime/ trajectory analysis was carried out by Monocle package under the Seurat condition in R, and cell cluster distribution in each cell state (described in Fig. 7h) along the pseu-dotime trajectory of these CRC-infiltrating myeloid cells is shown (h). Kinetics of relative expression levels of indicated genes along the MDSC 'transition' pseudo-time are shown (i) . TAM: tumor-associated macrophage; Mono-DC: monocyte-derived DC.

(j) Similar pseudotime/ trajectory analysis was carried out by using another human CRC scRNA-seq data (GSE146771) and kinetics of expression levels of indicated genes along the reverse MDSC 'transition' pseudo-time are shown.

Data in (d) are expressed as means and are analyzed using paired two-tailed Student's *t*-test. Source data are provided in the Source Data file.

Supplementary Table 1. Clinical information of colorectal cancer patients in the study

disease	sample #	T	N	M	TNM stage	pathological grading	tumor size (cm)
CRC	001359	T2	N0	M0	I	moderately differentiated	5.5
CRC	001361	T4	N0	M0	IIB	moderately~poorly	2.5
CRC	001364	T4	N0	M0	IIB	moderately~poorly	7
CRC	001365	T4a	N1	M0	IIIB	poorly differentiated	3.5
CRC	001367	T4	N0	M0	IIB	moderately~poorly	4
CRC	001368	T4	N1	M0	IIIB	moderately differentiated	4
CRC	001369	T3	N1	M0	IIIB	moderately differentiated	5
CRC	001371	T3	N1	M1	IV	moderately differentiated	5
CRC	001372	T4	N0	M0	IIB	moderately differentiated	7
CRC	001374	T4	N0	M0	IIB	moderately differentiated	4
CRC	001375	T3	N1a	M0	IIIB	poorly differentiated	6
CRC	001377	T4	N1	M1	IV	moderately differentiated	2.5
CRC	001379	T4	N1	M1	IV	moderately differentiated	6.5
CRC	001380	T4	N2	M1	IV	moderately differentiated	3
CRC	001381	T4	N2	M1	IV	poorly differentiated	8
CRC	001382	T4	N0	M0	IIB	moderately differentiated	3.5
CRC	001383	T3	N0	M0	II(A)	moderately differentiated	4
CRC	001384	T3	N1	M0	IIIB	moderately differentiated	2.5
CRC	001385	T4a	N1b	M0	IIIB	moderately differentiated	3
CRC	001386	T4b	N0	M1	IVa	moderately differentiated	18
CRC	001387	T4b	N1a	M0	IIIC	moderately differentiated	5
CRC	001388	T3	N2	M0	IIIB/C?	moderately~poorly	4
CRC	001389	T3	N1	M0	IIIB	moderately differentiated	1.5
CRC	001395	T3	N0	M0	IIA	moderately differentiated	10
CRC	001397	T3	N0	M0	IIA	moderately differentiated	7
CRC	001399	T3	N0	M0	IIA	moderately~poorly	7.5
CRC	001406	T4	N2	M1	IV	moderately differentiated	5
CRC	001407	T3	N1	M0	IIIB	moderately differentiated	3.5
CRC	001408	T2	N0	M0	I	well differentiated	5
CRC	001409	T4	N2	M1	IV	moderately~poorly	12
CRC	001410	T2	N1	M0	IIIA	moderately differentiated	2
CRC	001411	T4	N2	M0	IIIC	moderately differentiated	4
CRC	001412	T4	N0	M0	IIB	well~moderately	3.5
CRC	001413	T2	N0	M0	I	moderately differentiated	4.5
CRC	001414	T3	N2	M1	IV	moderately differentiated	4
CRC	001416	T4	N1	M0	IIIB/C?	moderately differentiated	7
CRC	001417	T4	N1	M0	IIIB/C?	moderately~poorly	7
CRC	001418	T3	N0	M0	II(A)	moderately differentiated	6
CRC	001419	T3	N3	M1	IV	moderately~poorly	5
CRC	001421	T4	N1	M0	IIIB/C?	moderately differentiated	4
CRC	001422	T3	N1	M0	IIIB	moderately differentiated	6
CRC	001423	T3	N1	M0	IIIC(B)	moderately differentiated	2.5
CRC	001424	T4	N2	M0	IIIC	poorly differentiated	10
CRC	001425	T4	N2	M0	IIIC	well~moderately	4
CRC	001427	T3	N0	M0	IIA	moderately differentiated	2.5
CRC	001428	T4	N0	M0	IIB	moderately differentiated	4
CRC	001429	T4	N0	M0	IIB	mucinous adenocarcinoma	2.5
CRC	001431	T2	N0	M0	I	moderately differentiated	8
CRC	001432	T4	N1	M1	IV	moderately differentiated	3
CRC	001433	T3	N2	M0	IIIC	poorly differentiated	7
CRC	001435	T3	N2	M0	IIIC	mucinous adenocarcinoma	4
CRC	001439	T3	N0	M1	IV	moderately differentiated	4
CRC	001441	T4	N0	M0	IIB	moderately differentiated	3
CRC	001443	T3	N0	M0	IIA	moderately~poorly	3.5
CRC	001444	T3	N2	M1	IV	moderately differentiated	4.5
CRC	001445	T4	N0	M0	IIB	moderately differentiated	4
CRC	001450	T4	N0	M0	IIB	moderately differentiated	9
CRC	001451	T4	N1	M0	IIIB/C?	well~moderately	4
CRC	001452	T4	N0	M0	IIB	moderately differentiated	7
CRC	001453	T4	N0	M0	IIB	moderately differentiated	9
CRC	001456	T4	N0	M0	IIB	mucinous adenocarcinoma	6
CRC	001460	T4	N1	M0	IIIB	moderately differentiated	6
CRC	001462	T3	N0	M0	IIB(A)	moderately~poorly	7
CRC	001463	T4	N2	M1	IV	poorly differentiated	7
CRC	001466	T4	N0	M0	IIB	moderately differentiated	5
CRC	001474	T2	N2b	M0	IIIB	moderately differentiated	3.5
CRC	001475	T4	N2	M1	IV	poorly differentiated	4.5
CRC	001476	T4a	N1	M0	IIIB	moderately differentiated	4

Supplementary Table 2. Primers used in Real-time PCR in the study

	Gene name	*Forward*	*Reverse*
mouse	**Clec7a**	GACTTCAGCACTCAAGACATCC	TTGTGTCGCCAAAATGCTAGG
	**Il1b**	CAACCAACAAGTGATATTCTCCATG	GATCCACACTCTCCAGCTGCA
	**Il6**	GAGGATACCACTCCCAACAGACC	AAGTGCATCATCGTTGTTCATACA
	**Il18**	ACTGTACAACCGCAGTAATACGC	AGTGAACATTACAGATTTATCCC
	**Il18bp**	CCTACTTCAGCATCCTCTACTGG	AGGGTTTCTTGAGAAGGGGAC
	**Il22ra2**	CATTGCCTTCTAGGTCTCCTCA	CCTGCTTGCCAGTGCAAAAT
	**Ptgs1**	ATGAGTCGAAGGAGTCTCTCG	GCACGGATAGTAACAACAGGGA
	**Ptgs2**	TTCAACACACTCTATCACTGGC	AGAAGCGTTTGCGGTACTCAT
	**Ptges2**	CCTCGACTTCCACTCCCTG	TGAGGGCACTAATGATGACAGAG
	**Ptges3**	TGTTTGCGAAAAGGAGAATCCG	CCATGTGATCCATCATCTCAGAG
	**Adh1**	GCAAAGCTGCGGTGCTATG	TCACACAAGTCACCCCTTCTC
	**Aldh1a1**	ATACTTGTCGGATTTAGGAGGCT	GGGCCTATCTTCCAAATGAACA
	**Aldh1a7**	ACTTGGAAGTTAGGCCCTGC	TGTGAAGGACACTTTGTCGATG
	**Rdh7**	TGGGTCGAGTGTCTTTGTGTG	AACCCGCCAGGCTCTATGATA
	**Eef1a1**	CAACATCGTCGTAATCGGACA	GTCTAAGACCCAGGCGTACTT
	**Eef2**	TGTCAGTCATCGCCCATGTG	CATCCTTGCGAGTGTCAGTGA
	**Pabpc1**	CAAGCCAGTACGCATCATGTG	TGCTTCCTGTGTTTCAAAGTGT
	**Lgals9**	ATGCCCTTTGAGCTTTGCTTC	AACTGGACTGGCTGAGAGAAC
	**H2-Ab1**	AGCCCCATCACTGTGGAGT	GATGCCGCTCAACATCTTGC
	**Ki67**	T ATCATTGACCGCTCCTTTAGGT	GCTCGCCTTGATGGTTCCT
	**Vegfa**	GCACATAGAGAGAATGAGCTTCC	CTCCGCTCTGAACAAGGCT
	**Ccnd1**	GCGTACCCTGACACCAATCTC	CTCCTCTTCGCACTTCTGCTC
	**Myc**	ATGCCCCTCAACGTGAACTTC	CGCAACATAGGATGGAGAGCA
	**Arg1**	CTCCAAGCCAAAGTCCTTAGAG	AGGAGCTGTCATTAGGGACATC
	**Nos2**	GTTCTCAGCCCAACAATACAAGA	GTTCTCAGCCCAACAATACAAGA
	**Itgam**	ATGGACGCTGATGGCAATACC	TCCCCATTCACGTCTCCCA
	**Foxp3**	CCCATCCCCAGGAGTCTTG	ACCATGACTAGGGGCACTGTA
	**Gapdh**	TTCACCACCATGGAGAAGGC	GGCATGGACTGTGGTCATGA
human	**CD33**	GGTGTGACTACGGAGAGAACC	GGTAGGGTGGGTGTCATTCC
	**HLA-DRA**	ATACTCCGATCACCAATGTACCT	GACTGTCTCTGACACTCCTGT
	**ITGAM**	GCCTTGACCTTATGTCATGGG	CCTGTGCTGTAGTCGCACT
	**CLEC7A**	GGAAGCAACACATTGGAGAATGG	CTTTGGTAGGAGTCACACTGTC
	**IL22RA2**	TGTTGGGGTACTCAAGAACTCT	CCCTCCCGTAATAAGGTTCCTG
	**PLA2G2A**	GAAAGGAAGCCGCACTCAGTT	CAGACGTTTGTAGCAACAGTCA
	**PTGS2**	CTGGCGCTCAGCCATACAG	CGCACTTATACTGGTCAAATCCC
	**PTGES**	TCCTAACCCTTTTGTCGCCTG	CGCTTCCCAGAGGATCTGC
	**GAPDH**	TGTGGGCATCAATGGATTTGG	ACACCATGTATTCCGGGTCAAT
