# Supplementary material for: Blocking Dectin-1 prevents colorectal tumorigenesis by suppressing prostaglandin E2 production in myeloid-derived suppressor cells and enhancing IL-22 binding protein expression
Source: Nat Commun. 2023 Mar 17;14:1493. doi: 10.1038/s41467-023-37229-x (PMC10023663; doi:10.1038/s41467-023-37229-x)
Supplement: Supplementary file 3 — Reporting Summary [file 41467_2023_37229_MOESM3_ESM.pdf]

nature portfolio

Corresponding author(s): Ce Tang

Last updated by author(s): Mar 3, 2023

Reporting Summary

Nature Portfolio wishes to improve the reproducibility of the work that we publish. This form provides structure for consistency and transparency in reporting. For further information on Nature Portfolio policies, see our Editorial Policies and the Editorial Policy Checklist.

Statistics

For all statistical analyses, confirm that the following items are present in the figure legend, table legend, main text, or Methods section.

n/a	Confirmed	
☐	☒	The exact sample size (*n*) for each experimental group/condition, given as a discrete number and unit of measurement
☐	☒	A statement on whether measurements were taken from distinct samples or whether the same sample was measured repeatedly
☐	☒	The statistical test(s) used AND whether they are one- or two-sided<br>*Only common tests should be described solely by name; describe more complex techniques in the Methods section.*
☒	☐	A description of all covariates tested
☐	☒	A description of any assumptions or corrections, such as tests of normality and adjustment for multiple comparisons
☐	☒	A full description of the statistical parameters including central tendency (e.g. means) or other basic estimates (e.g. regression coefficient) AND variation (e.g. standard deviation) or associated estimates of uncertainty (e.g. confidence intervals)
☐	☒	For null hypothesis testing, the test statistic (e.g. *F*, *t*, *r*) with confidence intervals, effect sizes, degrees of freedom and *P* value noted<br>*Give P values as exact values whenever suitable.*
☒	☐	For Bayesian analysis, information on the choice of priors and Markov chain Monte Carlo settings
☒	☐	For hierarchical and complex designs, identification of the appropriate level for tests and full reporting of outcomes
☒	☐	Estimates of effect sizes (e.g. Cohen's *d*, Pearson's *r*), indicating how they were calculated

Our web collection on statistics for biologists contains articles on many of the points above.

Software and code

Policy information about availability of computer code

Data collection	CellQuest Pro 5.1 and FACSDiva 8.0 software (BD Biosciences) were used to collect data by flow cytometry. CFX Manager 2.1 Software (Bio-Rad) was used to collect data of real-time qPCR.
Data analysis	We used GraphPad Prism v8 & 9.0 for log rank (Mantel-Cox) test and one/two-way ANOVA followed by multiple-comparisons test, and Microsoft 365 Excel or SPSS 24.0 for Student's t test and Mann-Whitney U test. For CRC patient study, correlation was analyzed by the Spearman's correlation test and statistical analysis was performed using the R software v4.0.3. FlowJo v10.0 & v10.1. FACS software (BD) were used to analyze data obtained from Flow Cytometer. CFX Manager 2.1 (Bio-Rad) and Microsoft 365 Excel were used to analyze the real-time qPCR data. We used R 4.1.3 and Rstudio Desktop v2022.02.3 for the single cell RNA-seq analysis.

For manuscripts utilizing custom algorithms or software that are central to the research but not yet described in published literature, software must be made available to editors and reviewers. We strongly encourage code deposition in a community repository (e.g. GitHub). See the Nature Portfolio guidelines for submitting code & software for further information.

Data

Policy information about availability of data

All manuscripts must include a data availability statement. This statement should provide the following information, where applicable:

- Accession codes, unique identifiers, or web links for publicly available datasets
- A description of any restrictions on data availability
- For clinical datasets or third party data, please ensure that the statement adheres to our policy

Barcodes, features and matrix files for scRNA-seq analysis and raw sequencing files for bulk RNA-seq analysis have been deposited in GEO (http://www.ncbi.nlm.nih.gov/geo/) with the accession number GSE221206. The publicly available data of single cell RNA sequencing used in this study are also available in the GEO database under accession code GSE196054, GSE178318 and GSE146771. All other relevant data supporting the findings of this study are available within the Article, in the Supplementary Information and in Source data. Source data are provided with this paper.

Human research participants

Policy information about studies involving human research participants and Sex and Gender in Research.

Reporting on sex and gender	We conventionally collected intestinal tumor samples from CRC patients after the tumorectomy. Human biospecimens from both male and female patients were collected.
Population characteristics	We conventionally collected intestinal tumor samples from CRC patients after the tumorectomy, ignoring these patients' age or genders. All CRC patients were Chinese yellow race and the detailed informations of their current diagnosis and treatment categories were provided in Supplementary Information. Two healthy volunteers who provided PBMCs are between 29-32 years old, male, Chinese yellow race and have no past medical history.
Recruitment	Human biospecimens were collected after the conventional surgical operation from CRC patients autonomously came to the hospital. Before the collection of human samples from CRC patients, written informed consent was obtained from each patient. Healthy volunteers were recruited by announcing the recruitment information in the institute of Precision Medicine in the affiliated hospital of Sun Yat-sen University, and after the written informed consent was obtained from each volunteers, PBMCs were harvested by medical doctors.
Ethics oversight	All experiments by using human biospecimens were carried out following the institutional ethical regulations and guidelines under the protocols approved by the Committee for Clinical Investigation of the First Affiliated Hospital, Sun Yat-sen University (approval number: IIT-2021-654)

Note that full information on the approval of the study protocol must also be provided in the manuscript.

Field-specific reporting

Please select the one below that is the best fit for your research. If you are not sure, read the appropriate sections before making your selection.

[x] Life sciences [ ] Behavioural & social sciences [ ] Ecological, evolutionary & environmental sciences

For a reference copy of the document with all sections, see nature.com/documents/nr-reporting-summary-flat.pdf

Life sciences study design

All studies must disclose on these points even when the disclosure is negative.

Sample size	We referred prior experiment of our laboratory to predetermine sample size that proper to identify differences between groups, statistical analyses and reproducibility. For mouse in vivo experiments and cell in vitro experiments, sample sizes were chosen based on the basis of our previous publications and the literature in the field, without prior power analysis. We usually used more than 4 mice/group or more than 3 wells/group, and in this case, we should have 80% power to detect a difference in means between two or among several groups at a significance level of 0.05 to ensure the statistically significant difference to be obtained from two-tailed Student's t-test or from one/two-way ANOVA followed by multiple-comparisons test. We usually collected more than 8 samples/group to obtain the statistically significant difference by Pearson's or Spearman's correlation (r) test. We usually collected samples as many as possible (usually more than 10) to obtain the significant difference by Mantel-Cox log-rank test. Each sample was prepared from biologically independent mouse or human and all in vivo or in vitro experiments were replicated at least two times. However, we also tried to minimize the animal number to conform to the 3R guidelines for the animal experiments. Exact numbers of animals used in individual experiments are indicated in figure legends. Sample size were referenced previously published article (https://www.nature.com/articles/s41590-018-0134-y, https://doi.org/10.1016/j.chom.2015.07.003)
Data exclusions	There were no excluded samples or data from the analysis in this study.
Replication	All in vivo or in vitro experiments were replicated at least two times. For each experiment, all attempts at replication were successful. The exact number of replication were described in each figure legend.

Randomization	All mice were grouped by their genotype or treatment condition, and inside the each aimed or control group, mice were randomly chose from the same or different littermates with same sex and similar date of birth. For in vitro experiment, samples were organized into groups according to genotype, and before treatment, samples were randomly allocated to each group. We conventionally collected intestinal tumor samples from CRC patients after the tumorectomy, ignoring these patients' age or genders.
Blinding	As the Dectin-1 gene-deficient mice we generated were preliminarily analyzed in other studies, and to definitely identify/distinguish the differences of immune functions among these genes, it was not necessary for us to be blinded to group allocation because this method is usually appropriate for the first investigation of a function-totally-unknown gene. But for the survival observation of ApcMin, ApcMin-Clec7a and ApcMin-Clec7a-Il22ra2KO mice, survival time of each mouse was determined by the moribund condition which was assessed and announced by staffs works in the animal facility, who didn't know the information of groups assigned. Therefore the survival experiment was single-blinded.

Reporting for specific materials, systems and methods

We require information from authors about some types of materials, experimental systems and methods used in many studies. Here, indicate whether each material, system or method listed is relevant to your study. If you are not sure if a list item applies to your research, read the appropriate section before selecting a response.

Materials & experimental systems

n/a	Involved in the study
☐	☒ Antibodies
☒	☐ Eukaryotic cell lines
☒	☐ Palaeontology and archaeology
☐	☒ Animals and other organisms
☒	☐ Clinical data
☒	☐ Dual use research of concern

Methods

n/a	Involved in the study
☒	☐ ChIP-seq
☐	☒ Flow cytometry
☒	☐ MRI-based neuroimaging

Antibodies

Antibodies used

Antibodies against mouse CD4 (GK1.5, Cat# 100411), CD8a(53-6.7, Cat# 100707), TCRgd (GL3, Cat# 118105), CD45 (30-F11, Cat# 103115, 103113), CD19 (6D5, Cat# 115505), CD3e (KT3.1.1, Cat# 155607), Ly6C (HK1.4, Cat# 128007, 128025), Ly6G (1A8, Cat# 127605, 127633), CD11b (M1/70, Cat# 101215), CD11c (N418, Cat# 117321), I-A/I-E (M5/114.15.2, Cat# 107613, 107627), CD103 (2E7, Cat# 121405), IgG1 (RMG1-1, Cat# 406607), NOS2 (W16030C, Cat# 696805), IFN-g (XMG1.2, Cat# 505805) , IL-17 (TC11-18H10.1, Cat# 506917), Foxp3 (MF-14, Cat# 126403), CD326 (G8.8, Cat# 118203), Thy1.2 (30-H12, Cat# 105304) and anti-human CD33 (HIM3-4, Cat# 303303), CD14 (M5E2, Cat# 301807), CD15 (W6D3, Cat# 323005), HLA-DR (L243, Cat# 307645), CD3e (HIT3a, Cat# 300305) and CD8a (SK1, Cat# 344721) were purchased from Biolegend (San Diego, U.S.A.). Antibody against mouse Dectin-1 (2A11, Cat# ab21646) was purchased from Abcam (Cambridge, UK). Anti-mouse IL-22BP polyclonal antibody was purchased from R&D systems (Cat# IC2376A, Biotechne, Minnesota, U.S.A). 7-AAD (7-amino-actinomycin D) staining solution were obtained from eBioscience (Cat# 00-6993-50, San Diego, U.S.A.). The anti-mouse CD16/CD32 (2.4G2, anti-FcgRII/III) mAb was obtained from Tonbo Biosciences (Cat# 70-0161-U500, San Diego, CA, U.S.A.). Anti-mouse IL-18 antibody (Cat# 210-401-323, Rockland Immunochemicals, Inc, Philadelphia, PA, USA) was used for in vitro culture. Mouse IL-22BP Antibody (Bio-techne, cat# AF2376), Human/Mouse p23/PTGES3 Antibody (Bio-techne, cat# MAB100391). Antibody against human Dectin-1 (Cat# ab140039, Rabbit polyclonal to human and Rat Dectin-1, Abcam Inc, Cambridge, UK) and HRP-conjugated anti-rabbit IgG (Cat# GB23303, Servicebio co. Wuhan, China) were used for CRC tumor tissue IHC staining. Human/Mouse COX-2 Antibody (Bio-techne, cat# AF4198-SP), Human/Mouse COX-1 Antibody (596002, Bio-techne, cat# MAB37401), THETM beta-Actin mouse Antibody (2D1D10, GenScript, cat# A00702-100), HRP-conjugated Affinipure Donkey Anti-Goat IgG (H+L) (Proteintech, cat# SA00001-3), HRP-conjugated Affinipure Rabbit Anti-Sheep IgG (H+L) (Proteintech, cat# SA00001-16), Goat Anti-Mouse IgG Antibody (H&L) [HRP] pAb (GenScript, cat# A00160) were used for Western blot analysis.

Validation

All antibodies used in this manuscript have been confirmed by the authors for their species, cross-reaction and application on manufacturers'websites. All antibodied were confirmed by the authors for their applicability in the present study. Specific validation information for each commerciall antibody is described in the related website:

APC anti-mouse CD4 Antibody (GK1.5) https://www.biolegend.com/en-us/products/apc-anti-mouse-cd4-antibody-245
PE anti-mouse CD8a Antibody (53-6.7) https://www.biolegend.com/en-us/products/pe-anti-mouse-cd8a-antibody-155
FITC anti-mouse TCR γ/δ Antibody (GL3) https://www.biolegend.com/en-us/products/fitc-anti-mouse-tcr-gamma-delta-antibody-2420
APC/Cyanine7 anti-mouse CD45 Antibody (30-F11) https://www.biolegend.com/en-us/products/apc-cyanine7-anti-mouse-cd45-antibody-2530
PE/Cyanine7 anti-mouse CD45 Antibody (30-F11) https://www.biolegend.com/en-us/products/pe-cyanine7-anti-mouse-cd45-antibody-1903
FITC anti-mouse CD19 Antibody (6D5) https://www.biolegend.com/en-us/products/fitc-anti-mouse-cd19-antibody-1528
PE anti-mouse CD3ε Antibody (KT3.1.1) https://www.biolegend.com/en-us/products/pe-anti-mouse-cd3e-antibody-16474
PE anti-mouse Ly-6C Antibody (HK1.4) https://www.biolegend.com/en-us/products/pe-anti-mouse-ly-6c-antibody-4904
APC/Cyanine7 anti-mouse Ly-6C Antibody (HK1.4) https://www.biolegend.com/en-us/products/apc-cyanine7-anti-mouse-ly-6c-antibody-6758
FITC anti-mouse Ly-6G Antibody (1A8) https://www.biolegend.com/en-us/products/fitc-anti-mouse-ly-6g-antibody-4775

Brilliant Violet 510™ anti-mouse Ly-6G Antibody (1A8) https://www.biolegend.com/en-us/products/brilliant-violet-510-anti-mouse-ly-6g-antibody-9121
PE/Cyanine7 anti-mouse/human CD11b Antibody (M1/70) https://www.biolegend.com/en-us/products/pe-cyanine7-anti-mouse-human-cd11b-antibody-1921
Pacific Blue™ anti-mouse CD11c Antibody (N418) https://www.biolegend.com/en-us/products/pacific-blue-anti-mouse-cd11c-antibody-3864
APC anti-mouse I-A/I-E Antibody (M5/114.15.2) https://www.biolegend.com/en-us/products/apc-anti-mouse-i-a-i-e-antibody-2488
APC/Cyanine7 anti-mouse I-A/I-E Antibody (M5/114.15.2) https://www.biolegend.com/en-us/products/apc-cyanine7-anti-mouse-i-a-i-e-antibody-5966
PE anti-mouse CD103 Antibody (2E7) https://www.biolegend.com/en-us/products/pe-anti-mouse-cd103-antibody-3574
PE anti-mouse IgG1 Antibody (RMG1-1) https://www.biolegend.com/en-us/products/pe-anti-mouse-igg1-6494
PE anti-Nos2 (iNOS) Antibody (W16030C) https://www.biolegend.com/en-us/products/pe-anti-nos2-inos-antibody-19910
FITC anti-mouse IFN-γ Antibody (XMG1.2) https://www.biolegend.com/en-us/products/fitc-anti-mouse-ifn-gamma-antibody-995
Pacific Blue™ anti-mouse IL-17A Antibody (TC11-18H10.1) https://www.biolegend.com/en-us/products/pacific-blue-anti-mouse-il-17a-antibody-4145
PE anti-mouse FOXP3 Antibody (MF-14) https://www.biolegend.com/en-us/products/pe-anti-mouse-foxp3-antibody-4660
Biotin anti-mouse CD326 (Ep-CAM) Antibody (G8.8) https://www.biolegend.com/en-us/products/biotin-anti-mouse-cd326-ep-cam-antibody-4725
Biotin anti-mouse CD90.2 (Thy1.2) Antibody (30-H12) https://www.biolegend.com/en-us/products/biotin-anti-mouse-cd90-2-thy1-2-antibody-103
FITC anti-human CD33 Antibody (HIM3-4) https://www.biolegend.com/en-us/products/fitc-anti-human-cd33-antibody-726
APC anti-human CD14 Antibody (M5E2) https://www.biolegend.com/en-us/products/apc-anti-human-cd14-antibody-793
PE anti-human CD15 (SSEA-1) Antibody (W6D3) https://www.biolegend.com/en-us/products/pe-anti-human-cd15-ssea-1-antibody-3701
Brilliant Violet 510™ anti-human HLA-DR Antibody (L243) https://www.biolegend.com/en-us/products/brilliant-violet-510-anti-human-hla-dr-antibody-8008
FITC anti-human CD3 Antibody (HIT3a) https://www.biolegend.com/en-us/products/fitc-anti-human-cd3-antibody-751
APC anti-human CD8 Antibody (SK1) https://www.biolegend.com/en-us/products/apc-anti-human-cd8-antibody-6531
FITC Anti-Dectin-1 antibody (2A11) https://www.abcam.com/fitc-dectin-1-antibody-2a11-ab21646.html
Mouse IL-22BP APC-conjugated Antibody (polyclonal) https://www.rndsystems.com/cn/products/mouse-il-22bp-apc-conjugated-antibody_ic2376a
Anti-mouse CD16/CD32 (2.4G2) https://www.biocompare.com/9776-Antibodies/9247042-Purified-Anti-Mouse-CD16-CD32-2-4G2/
Mouse IL-18 Antibody (Polyclonal) https://www.rockland.com/categories/primary-antibodies/mouse-il-18-antibody-210-401-323/
Anti-Dectin-1 antibody (polyclonal) https://securedrtest.abcam.com/dectin-1-antibody-ab140039.html
Goat Anti-Rabbit IgG H&L (HRP) https://www.servicebio.com/goodsdetail?id=266
Human/Mouse COX-2 Antibody (Polyclonal) https://www.rndsystems.com/cn/products/human-mouse-cox-2-antibody_af4198
Human/Mouse COX-1 Antibody (596002) https://www.rndsystems.com/cn/products/human-mouse-cox-1-antibody-596002_mab37401
THE™ beta Actin Antibody, mAb, Mouse (2D1D10) https://www.genscript.com/antibody/A00702-THE_beta_Actin_Antibody_mAb_Mouse.html
HRP-conjugated Affinipure Donkey Anti-Goat IgG(H+L) https://www.ptglab.com/products/Peroxidase-conjugated-Affinipure-Donkey-Anti-Goat-IgG-H-L.htm
HRP-conjugated Affinipure Rabbit Anti-Sheep IgG(H+L) https://www.ptglab.com/products/Peroxidase-conjugated-Affinipure-Rabbit-Anti-Sheep-IgG-H-L.htm
Goat Anti-Mouse IgG Antibody (H&L) [HRP], pAb https://www.genscript.com/antibody/A00160-Goat_Anti_Mouse_IgG_Antibody_H_L_HRP_pAb_.html

Animals and other research organisms

Policy information about studies involving animals; ARRIVE guidelines recommended for reporting animal research, and Sex and Gender in Research

Laboratory animals	Clec7a−/− mice were used after backcrossing for 9 generations to C57BL/6J. ApcMin/+ mice, kindly provided by Dr. Ryo Abe, Tokyo University of Science, were crossed with Clec7a−/− mice to generate ApcMin/+Clec7a−/− mice. C57BL/6J mice which were originally purchased from Sankyo Lab Service (Saitama, Japan) for Clec7a−/− mouse backcrossing, and their offspring were maintained in the same animal room as that Clec7a−/− mice were housed and used as the control. GF WT mice were purchased from Sankyo Lab Service. GF Clec7a−/− mice were generated by taking out sterile babies from the uterus of a SPF full-term pregnant female by cesarean section and transferring these babies in the care of 2 to 3 GF WT mothers that were nursing their recently delivered litters in a GF isolator. Age- and sex-matched WT mice were separately housed or co-housed with Clec7a−/− mice after weaning at 4 weeks old. All mice were kept under specific pathogen-free conditions with gamma-ray sterilized normal diet, acidified (0.002 N HCl, pH 2.5) tap water, and autoclaved wooden chip bed in environmentally controlled clean rooms. Experimental animal facilities of the Center for Animal Disease Models, Research Institute for Biomedical Sciences, Tokyo University of Science, and of Zhongshan School of Medicine, Sun Yat-sen University usually provide 12h/12h light/dark cycle at 23-24 degrees Celsius and 40-50% of humidity range for mouse keeping. All mice used in the present study are on C57BL/6J background. Mice with 8-10 weeks old were used. Both male and female mice were used.
Wild animals	This study did not involve wild animals.
Reporting on sex	Both male and female mice were used in the present study, as mice with both sex exhibited same phenotypes and similar tendency of differences between WT and gene-deficient hosts.

Field-collected samples	This study did not involve samples collected from the field.
Ethics oversight	All animal experiments were carried out following the institutional ethical regulations and guidelines and with protocols approved by the Institutional Animal Care and Use Committee of the Tokyo University of Science, and by the Experimental Animal Manage and Use Committee of Sun Yat-sen University (approval number 2020000113, 2021001577).

Note that full information on the approval of the study protocol must also be provided in the manuscript.

Flow Cytometry

Plots

Confirm that:

- [x] The axis labels state the marker and fluorochrome used (e.g. CD4-FITC).
- [x] The axis scales are clearly visible. Include numbers along axes only for bottom left plot of group (a 'group' is an analysis of identical markers).
- [x] All plots are contour plots with outliers or pseudocolor plots.
- [x] A numerical value for number of cells or percentage (with statistics) is provided.

Methodology

Sample preparation	Colonic tumor/ non-tumor tissue-infiltrating cells were prepared as follows. Briefly, tissue pieces were cut into 1 mm slices, then they were shaken for 40 min in Hanks' Balanced Salt Solution (HBSS) containing 3 mM EDTA at 37oC. Intra-epithelial lymphocytes and colonic epithelial cells in the culture supernatant were discarded, and the gut slices were washed twice with HBSS. Then, they were shaken at 37oC for 120 min in RPMI containing 10% FBS and 1% streptomycin+penicillin, 200 U/ml collagenase (C2139; Sigma-Aldrich), and 5 U/ml Dnase 1 (Sigma-Algrich). After the incubation, the samples were vortexed for 10 sec, and single cell suspension was harvested after sterile gauze-filtration. To prepare lymphocytes, tissue-infiltrating cells were further purified on a 45%/66.6% discontinuous Percoll (Pharmacia, Uppsala, Sweden) gradient at 2200 rpm for 20 min.
Instrument	Flow cytometry was perfromed on an BD FACSCanto II (BD Biosciences) at Tokyo University of Science.
Software	CellQuest Pro 5.1 and FACSDiva 8.0 software (BD Biosciences) were used to collect the FACS data. FlowJo 10.0 & 10.1 FACS software (BD) were used to analyze data obtained from Flow Cytometer.
Cell population abundance	Flow cytometry analysis were performed on single cell isolation from intestinal tumor or non-tumor tissues, and data presented represent the percentages for each population in intestinal leukocytes, including myeloid-derived cells and lymphocytes.
Gating strategy	Usually, we firstly gated the cell cluster located on the left bottom of FSC-SSC dot panel (region from 30K~120K of FSC axis and 10K~100K of SSC axis) with the elliptic gate, and then gated cells on FSC-W x FSC-A to get the single cells. We then selected the live cells by gating on 7AAD-negative cells with rectangle gate. To analyze sub-populations within the leukocytes, we further gated on CD45+ population in histogram.

- [x] Tick this box to confirm that a figure exemplifying the gating strategy is provided in the Supplementary Information.
